# Forensic skeletal and molecular anthropology face to face: Combining expertise for identification of human remains

**DOI:** 10.1111/nyas.15398

**Published:** 2025-07-10

**Authors:** Elena Pilli, Andrea Palamenghi, Cristina Cattaneo

**Affiliations:** ^1^ IRIS, Dipartimento di Biologia Università degli Studi di Firenze Firenze Italy; ^2^ LABANOF, Dipartimento di Scienze Biomediche per la Salute Università degli Studi di Milano Milano Italy

**Keywords:** biological profile, DNA, forensic anthropology, molecular anthropology, personal identification, skeleton

## Abstract

Forensic anthropology (FA) has grown significantly as a well‐established and independent discipline dedicated to the examination and identification of human remains in medicolegal and humanitarian contexts. When soft tissues are highly decomposed, skeletal analysis often provides more reliable data for reconstructing biological profiles and determining identity. The increasing number of unidentified decedents in both domestic and humanitarian scenarios highlights the need for forensic anthropologists. In this context, molecular anthropology (MA) could support FA by offering additional tools for forensic identification, although collaboration between these two fields remains uncommon. Drawing from the authors’ experience, this review emphasizes the value of collaboration to enhance case resolution. This review shows that when FA encounters limitations, MA may provide critical insight to address unanswered questions. Although the full integration of FA and MA has yet to be realized, combining the strengths of both fields allows for the creation of more comprehensive biological profiles, thus significantly improving the chances of identifying unknown remains. This interdisciplinary approach broadens FA's scope and drives the development of innovative techniques and methodologies, advancing the pursuit of truth and justice.

## Graphical abstract

Forensic anthropology has grown into a vital discipline for the identification of human remains when conventional methods fail. Molecular anthropologists, skilled in analyzing degraded DNA, can significantly aid in identification and profiling. This review emphasizes the importance of collaboration between these fields, showcasing how their combined expertise enhances positive identification and the creation of comprehensive biological profiles.

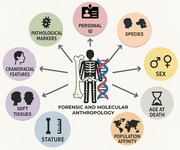

## INTRODUCTION

Personal identification of human remains is an increasingly urgent forensic task due to the growing number of unidentified decedents in both domestic and humanitarian contexts.^1^, ^2^, ^3^, ^4^, ^5^, ^6^ It is a multifaceted process aimed at determining the status or condition of missing or unidentified individuals. Identification is essential for issuing a death certificate, which legally confirms an individual's death and is critical for humanitarian, criminal, civil, administrative, and ethical purposes. Moreover, it helps prevent the distress of ambiguous loss for the living, a state of uncertainty about whether a loved one is dead or alive, which often results in psychological and mental health issues.^7^, ^8^, ^9^, ^10^

Among the numerous scientific fields that aid in forensic identification, anthropology plays a significant role. Originally born as a branch of biological anthropology, forensic anthropology (FA)—usually referred to as forensic skeletal anthropology—has developed into an independent discipline with its own methods and standards, providing vital support to coroners, police, and legal professionals by aiding in the recovery, analysis, and interpretation of evidence derived from decomposed or skeletal remains. Over time, FA applications branched out to assist in diverse medicolegal issues beyond the skeleton, including assessment of age^11^, ^12^ and violence,^13^, ^14^ gait analysis,^15^ and personal identification^16^, ^17^ of living individuals. Moreover, FA has become crucial in criminal investigations, civil cases, locating missing persons, disaster victim identification (DVI),^18^, ^19^, ^20^, ^21^ and addressing humanitarian and human rights violations.^22^, ^23^

As another branch of biological anthropology, molecular anthropology (MA) also developed in the 20th century with the rediscovery of the fundamental laws of genetics and the discovery of the DNA structure in the 1950s. Significant advancements in molecular biology also contributed to the development of this new field. Furthermore, the collaboration between paleoanthropologists and molecular biologists resulted in the creation of new professional figures capable of extracting, amplifying, and sequencing DNA from ancient remains. Their expertise in addressing and solving daily challenges in analyzing complex biological material characterized by various issues, including DNA degradation, could also be utilized in forensics for personal identification. In the context of identifying human remains, FA and MA play a critical role in a forensic scenario, and both become essential when dealing with the identification of skeletal remains.

The current migration crisis as well as domestic crime scenarios are, however, strongly challenging forensic scientists in the mere task of identifying human remains. In this perspective, the general belief is that FA and MA are to be kept as separate entities with limited interplay and communication. Although MA—based on DNA analysis—is often considered technologically advanced and a self‐sufficient method for personal identification, our experience at the Universities of Milan and Florence (Italy) indicates that an integrated, multidisciplinary approach yields more robust and reliable results. This is particularly evident in complex cases where no DNA reference samples or familial data are available, or when the remains are highly fragmented and severely compromised. Drawing from their collaboration as Italian anthropologists in forensic and humanitarian cases involving human skeletal remains, the authors here present a comparative critical review of the several steps for the biological profile and positive identification. While the authors acknowledge that FA encompasses more than the analysis of skeletonized remains, this review specifically focuses on the tools available to forensic and molecular anthropologists for creating biological profiles and positively identifying skeletons in forensic contexts. For each step, FA and MA provide their lines of evidence, which sometimes can be intertwined in a comprehensive approach to complement and/or strengthen the analyses. This review examines how each discipline contributes on its own to the analysis of human skeletal remains and explores how they can complement each other—highlighting both their advantages and limitations. Furthermore, it underscores the importance of their synergistic application in forensic identification scenarios to address unresolved issues that produce undetermined results. This paper does not aim to provide a comparative study between different jurisdictions or academic protocols, nor guidelines or standard operating procedures. Rather, the objective is to contribute to the discourse of research and what the literature offers for investigating human remains in a multidisciplinary manner by reviewing existing literature and discussing potential advancements in the fields of FA and MA. Finally, this review proposes a novel perspective on how FA can work side by side with MA, hence combining expertise and bridging gaps in biological profiling and positive identification to strengthen the analysis of unidentified skeletal remains and, consequently, the discipline as a whole.

## PERSONAL OR POSITIVE IDENTIFICATION: THE ROLE OF FA

Achieving personal identification through morphological traits requires a detailed analysis of specific and unique characteristics observed in the unknown decedent (postmortem [PM] data), which are then compared to corresponding information from the presumed identity of the individual (antemortem [AM] data). This comparative process involves conventional radiographs, computed tomography (CT) or magnetic resonance imaging (MRI) scans, and medical records and is essential for establishing a reliable match and confirming identity. Personal identification is traditionally performed based on unquantified comparisons which have been in the past criticized especially in the rise of juridical demands for more sound and quantified evidence that proves the identity of unknown remains.^24^ While Daubert guidelines mandate that a method's application must be linked to an error rate, the Kumho ruling has addressed this by recognizing that there are fields where such quantification is not possible.^25^, ^26^, ^27^ Nevertheless, these rulings were intended as tools to assist judges in assessing the admissibility of methods used in courtroom testimony, rather than as strict standards for forensic anthropologists.^26^ Although identification is not typically the primary focus of FA in expert witness contexts—and full quantification of results provided by the discipline is not always attainable^26^, ^27^—our experience suggests that, from a practical standpoint, quantified outcomes are more easily understood by judicial authorities. Such quantification is often requested in court proceedings and enhances the perceived reliability of skeletal identification methods, particularly when compared to genetic analysis and other approaches that inherently provide statistical or probabilistic results. This viewpoint is also supported by the Forensic Anthropology Society of Europe (FASE).^28^ However, this practical need requires caution in correctly interpreting parameters that can be considered valid within the limit of the quality of the data. Moreover, traditional secondary identifiers have undergone a renewed evaluation, particularly in contexts where primary identifiers, such as DNA, are insufficient or cannot be relied upon alone. This is especially relevant in complex scenarios like the ongoing migration crisis or low resource settings where identifying individuals often requires a multifaceted approach that incorporates secondary traits to supplement or compensate for the limitations of primary methods.^5^, ^29^, ^30^, ^31^, ^32^, ^33^, ^34^

In this perspective, research has focused on the identifying potential of both soft tissue features (e.g., nevi) and skeletal traits (e.g., nonmetric or pathological characteristics).^35^, ^36^, ^37^, ^38^, ^39^, ^40^ Studies have yielded promising results, highlighting the value of these variables in assessing their uniqueness and utility for identification purposes. Further studies and improvements may, therefore, expand the toolkit available for forensic investigations, particularly in cases where traditional identifiers may be unavailable or inconclusive. The primary limitation of human identification based on skeletal evidence is the absence of a clear and unanimous consensus on the necessary quality and quantity of characters for personal identification.^41^, ^42^, ^43^ Discordant features can help resolve a case by excluding an identity, whereas numerous concordant traits, especially if common within the population, can only offer a judgment of compatibility or possibility. However, some rare traits within the population or group can provide a judgment of high probability or certainty.

The authors recognize that certain features are not widely used as personal identifiers and that the admissibility of evidence in court is strongly regulated in some countries, such as the United States.^25^ In contrast, Italian and other European courts have more variable standards regarding evidence admissibility^44^ for forensic anthropological issues. Although not universally recognized as a primary identifier,^45^ morphology of frontal sinuses in the comparison between AM and PM radiographic images is possibly the most broadly researched technique for personal identification on a skeletal basis.^46^, ^47^ Research on the topic is continuing to flourish on the use of three‐dimensional (3D) volumes from CT and MRI scans, which allow for a quantification of the comparison.^48^, ^49^, ^50^ Although the practical application of these novel tools to real cases still needs further validation, the approach has been successfully tested on sphenoid and maxillary sinuses,^51^, ^52^ lumbar vertebrae,^53^ teeth,^54^, ^55^ and palatal rugae.^56^ Forensic odontology also undeniably contributes to personal identification. Recent research topics include the analysis of the dental contour in 2D photographs^34^, ^57^ and 3D models,^58^ superimposition of 2D‐3D images^59^ that overcome the limitations of bidimensional methods related to the orientation of the structures under study. The potential of the trabecular pattern as a personal identifier has been demonstrated in several case studies,^60^, ^61^, ^62^, ^63^, ^64^ although research on this topic has experienced a setback.^61^ General morphology and skeletal variants have also been shown to be valuable structures in numerous forensic cases due to the remarkable number of features offered and widespread availability of thoracic X‐rays as AM evidence.^65^, ^66^, ^67^, ^68^, ^69^, ^70^, ^71^ Medical, orthopedic, cochlear, and prosthetic devices can be particularly useful, especially if they have a serial number that can trace the device back to a hospital, surgeon, or specific patient.^22^, ^72^, ^73^, ^74^ This is true even for burned^75^ or cremated remains.^76^

## PERSONAL OR POSITIVE IDENTIFICATION: THE ROLE OF MA

Personal identification through molecular methods requires DNA profiling and comparing unknown profiles with known reference profiles. One of the major challenges for forensic experts is recovering and analyzing DNA from highly degraded skeletal remains. Therefore, a thorough understanding of the challenges related to handling, extracting, and analyzing DNA from bone samples is essential. In this context, experts skilled in dealing with highly degraded DNA (ancient DNA) can play a crucial role in the positive identification of skeletal remains.^77^ Over the years, researchers in ancient DNA have adapted standard molecular techniques to address issues of DNA degradation, contamination, and the presence of inhibitors, ensuring that each step in the DNA analysis process is optimized to maximize DNA yield and minimize DNA loss.^78^ Although the focus of ancient DNA experts is not on identification, the valuable experience they have gained from working with such samples can be crucial for forensic activities. Of course, any approach used in forensic casework requires validation, but in the long run, the benefit of improved methods could be evident.

The first critical aspect of this process for positive personal identification is selecting the appropriate skeletal element, which depends on what is available. However, not all skeletal elements are equally effective in preserving DNA, which leads to the establishment of a hierarchy of preference.^79^ Molecular anthropologists have shown that petrous bone is the most suitable skeletal element for DNA analysis, even for forensic purposes.^80^, ^81^, ^82^ Additionally, ear ossicles have been identified as a viable alternative to petrous bones,^83^ and cementum is considered the best part of teeth for sampling DNA.^84^, ^85^

The next crucial step in DNA analysis involves cleaning the sample using both chemical (typically bleach) and physical methods, such as mechanically removing the bone surface with a rotary sanding tool and exposing the skeletal elements to ultraviolet (UV) radiation. The hard tissues must then be pulverized to dissolve the organic and inorganic portions of the tissue by physically breaking down bone/tooth material. A minimally invasive approach^86^ is recommended to preserve as much of the bone structure as possible, ensuring precise and accurate sampling without damaging the bone structure and producing a fine powder. The quantity of bone or tooth powder utilized for DNA extraction differs among laboratories. While many forensic analysis protocols suggest using between 0.2 and 2.5 g of bone powder, current ancient DNA extraction techniques have shown great efficacy with as little as 0.05 g of powder.^87^ Additionally, the methods for DNA extraction can vary. Demineralization is a common preliminary step in numerous forensic laboratories before proceeding with DNA extraction. However, it has been observed that the ethylenediaminetetraacetic acid (EDTA) washing buffer used for demineralizing bone powder, and is subsequently discarded, contains a large amount of DNA. To fully break down hard material and enhance DNA yield, it is recommended to perform complete demineralization by adding a detergent and proteinase K to the EDTA.^88^, ^89^, ^90^ Although various DNA extraction methods are used by forensic laboratories, not all are equally effective for forensic investigations due to DNA degradation. When comparing ancient and forensic DNA extraction techniques,^91^, ^92^ Emery et al.^93^ and Xavier et al.^94^ demonstrated that the Dabney method is preferred in cases of significant DNA degradation or sample preservation issues, whether using classical or massively parallel sequencing approaches.

Additionally, to obtain reliable results, adherence to strict criteria to prevent contamination is mandatory. As proposed by the ancient DNA approach, all steps of DNA analysis from bone, teeth, and hair samples should be performed in dedicated laboratories where no other biological specimen, such as reference samples, blood, semen, or saliva samples, are analyzed to reduce/minimize and control contaminations.

## INTEGRATIVE APPROACH AND INTERDISCIPLINARY COLLABORATION

Positive identification represents the ultimate objective in the analysis of human remains, whether this is achieved through the examination of skeletal features or the application of molecular methods. Currently, molecular anthropologists hold a unique position as the only specialists capable of quantifying the strength of an identification through probabilities and likelihood ratios. In contrast, skeletal analysis relies heavily on qualitative assessments and lacks a robust statistical framework to support its conclusions. Nevertheless, when DNA fails, many morphological features, if used appropriately, remain reliable identifiers as they are already accepted by INTERPOL DVI practices^45^ and strongly emphasized by FASE.^28^ Recent advancements in research have begun to pave the way for the development of more quantitative methods in morphological analysis, offering a promising avenue for improving the reliability and objectivity of skeletal identifications.

Both FA and MA are intrinsically linked to the condition and preservation of the remains, as well as the availability and quality of AM reference material for comparison. Unfortunately, such reference material may be scarce or incomplete, posing significant challenges to the identification process. When neither skeletal data nor molecular evidence alone is sufficient to support a positive identification, a multidisciplinary approach that integrates the strengths of both fields can significantly enhance the overall accuracy and credibility of the findings. The combination of morphological and molecular evidence has the potential to create a synergistic effect, yielding a more comprehensive and definitive identification process.

This raises an important question: if FA were to develop quantitative methods and establish standardized thresholds for positive identification, how might this evidence be effectively combined with the probabilistic results produced by MA when one of these alone is not sufficient? What protocols and frameworks would be required to harmonize these two sources of evidence, ensuring that they complement each other when one of the two alone is not sufficient? This is a crucial challenge for future research.

## THE BIOLOGICAL PROFILE: THE ROLE OF FA AND MA FACE TO FACE

The biological profile is a comprehensive reconstruction of an individual's key biological characteristics derived from their skeletal remains. The main components of the biological profile include sex, age‐at‐death, population affinity, and stature. These pillars serve as investigative leads or a basis for an identification hypothesis, which must be confirmed through more individuating techniques. This set of four generic parameters can be expanded by including additional traits associated with soft tissue features—if preserved—that may further enhance the profile's completeness, as well as pathological markers that have been recognized as valuable contributors to implement the profile^95^ and serve as individualizing factors of identity.^96^ Before constructing the biological profile of an individual, however, determining the species, especially of fragmented remains, is essential.

### Species (human/nonhuman) identification

#### Forensic

The distinction between human or nonhuman origin of bones is of vital importance to initiate a judicial investigation. With complete bones, gross anatomy evaluated by macroscopic assessment of diagnostic features, especially at the articular surfaces, would be sufficient. When fragmented, histological analyses offer a valuable diagnostic tool. The microscopic configuration of human bone presents a well‐defined system that includes circumferential lamellar bone, primary osteons, resorption spaces, secondary osteons, and fragments of secondary osteons.^97^ Differences in this pattern have been documented, as nonhuman specimens exhibit a peculiar, layered arrangement of the osteon system known as plexiform bone or osteon banding (i.e., parallel rows of osteons or strips of nonlamellar bone), which is possibly the main discriminating feature in this analysis.^97^ However, caution about the osteon banding structure as a diagnostic factor has been suggested as infant or healing bones may also present this pattern.^98^ Morphological studies of the bone microstructure have increased so that it is possible to distinguish humans from several other species of mammals. Histomorphometric analyses can implement the results of the above‐mentioned approaches with quantitative data,^99^, ^100^, ^101^ especially when the human osteon pattern is shared with other species.^102^ Protein analysis is particularly effective as it delivers species‐specific results while requiring only a small amount of bone material.^103^, ^104^ However, its application may be somewhat limited due to the associated costs and the specialized expertise needed.^97^

#### Molecular

Molecular methods for species identification have been developed. As previously presented in Pilli et al.^105^, the primary molecular techniques used for identifying species for forensic relevance rely on mitochondrial DNA (mtDNA) markers. These markers are selected for forensic species identification due to their low variability within the same species (intraspecific variability) and their high variability between different species (interspecific variability), which allows for distinguishing individuals of different species. In traditional species identification of degraded samples, a short fragment of mtDNA genes is amplified and then sequenced using the Sanger method. The resulting sequence is compared and aligned with reference sequences stored in databases like GenBank and using tools such as BLAST. Molecular methods widely used for years may be considered an accurate tool and valid support for species identification when morphologically unidentifiable bones or bone fragments are the unique available evidence. In addition, a recent study^106^ highlighted the possibility to combine zooarchaeology by mass spectrometry (ZooMS) and ancient DNA methods to efficiently select human bone from the others via proteomic approach and improve the DNA yield via ancient DNA method in order to solve two old murder cases.

#### Integrative approach and interdisciplinary collaboration

Both macroscopic and microscopic bone analysis, as well as molecular techniques such as mtDNA analysis, can be used to identify human and nonhuman remains. These approaches complement each other. Macroscopic methods and gross anatomy are particularly effective when remains are well‐preserved and include diagnostic features, such as the ends of long bones. However, when remains are highly fragmented, further investigation at the microscopic level becomes essential. Molecular methods are especially valuable for degraded or fragmented samples, providing precise species identification through genetic markers. While both microscopic and molecular analyses are more time‐intensive than macroscopic methods, they are indispensable for accurately distinguishing between human and nonhuman fragments.

Cost‐efficiency is an important factor when selecting the appropriate methodology, particularly when dealing with a large number of samples. For heavily fragmented remains, it is crucial to preserve portions of the specimen whenever possible to allow for complementary analyses by both disciplines. Collaboration between forensic and molecular anthropologists significantly enhances species identification by combining morphological and genetic data, ultimately yielding more accurate and reliable results in forensic investigations.

### Sex estimation

#### Forensic

Determination of the skeletal sex from human remains is based on morphometric differences between males and females (sexual dimorphism). The conundrum of sexing juvenile skeletal remains is still unresolved. The use of the auricular surface's shape is suggested, reaching an accuracy rate of 80%.^107^, ^108^ However, accuracy rates as low as 36% have been recorded; therefore, this argument is still debated. Other traits include the subpubic concavity,^109^ although the method seems more reliable for late adolescents. Morphometric assessment of ilia from CT scans revealed that sex estimation is limitedly applicable prior to acetabular fusion.^110^ Measurements of limb bones from radiographs of individuals between birth and 12 years of age have yielded promising results with accuracy rates between 70% and 93%.^111^ Linear measurements of the bony labyrinth were recently suggested as an additional tool for sexing individuals under 16 years old.^112^

The pelvis, and especially pubic bones, is the most reliable region for sex estimation in adults. The unquantified assessment has been criticized,^113^ and further studies suggest scoring systems and equations that provide the probability of classification,^114^ with accuracy rates between 86.2% and 94.5%. Tests on different populations yielded 100% accuracy,^115^ or lower rates.^116^, ^117^ Although sex estimation of skeletons still seems to be pelvic and pubic‐centered (e.g., ventral arc, subpubic concavity, ischiopubic ramus), the literature acknowledges the experience bias of the observations.^118^, ^119^ Among the other traits, the greater sciatic notch was the most reliable, whereas the inferior and overall shape of the auricular surface was not to be used due to poor accuracy rates.^120^ The preauricular sulcus is reportedly more common in females than in males,^121^ although the reliability of this trait has been negatively^122^, ^123^ discussed. One CT scan study has provided statistical regression functions with a 99.2% accuracy rate, along with a considerate rate of indeterminate individuals.^124^ Diagnose Sexuelle Probabiliste (DSP) is a novel tool for a statistically supported sex estimation of the pelvis and considers metric variables of the innominate bone and is reported to have exceptional accuracy rates (100%). The second version of the method (DSP2)^125^ demonstrated that sexual metric dimorphism of the pelvis is population independent,^126^ hence it is a powerful tool that can be reliably applied to unknown individuals. DSP2 was tested on different skeletal groups with variable with generally satisfying results,^127^, ^128^, ^129^, ^130^ yet lower (85–96%) in terms of classification accuracy. DSP also has been demonstrated to work efficiently with optimal accuracy rates on virtual bone models.^131^, ^132^ Moreover, recent advancements in virtual anthropology on automatic landmarking and extraction of the variables maintain very low error rates, both for automated measurements and classification.^133^

Despite being a sexually dimorphic area, the cranium is a less reliable indicator of sex than the pelvis. Scoring systems, discriminant functions, and decision trees^134^, ^135^ provide morphological observations with statistical support, with variable results and sex bias^136^ (between 80% and 96% accuracy). Recent tests determined that mental eminence is the less reliable feature because of the poor inter‐rater agreement.^137^ Moreover, the literature suggests that population‐specific equations are needed to reliably apply and interpret the assessments. Tests on different populations (e.g., South Africans,^138^ Hispanics,^139^ and Italians^140^) have been carried out. Cranial measurements are also significant parameters, with the discriminant function program Fordisc,^141^ which allows extensive collection of craniometric data for comparative studies. Given the partially limited application of Fordisc outside of the United States, population‐specific studies^142^, ^143^, ^144^ are needed to tailor the use of the software on different groups.

Although the pelvis and cranium are generally considered the traditionally preferred elements for sex estimation,^145^, ^146^, ^147^ some authors argue that postcranial metric analysis of limb bones provides the second most reliable evidence after the pelvis and before the cranium.^148^, ^149^ Sectioning points from univariate and multivariate classification functions based on cranial and postcranial metrics were also developed with a correct classification rate of 88–90%.^149^ Notably, multivariate analysis of limb bones provides more accurate (up to 94%) sex estimation compared to the cranium.^149^ The proximal end of the femur has been widely investigated in several reports that have been exhaustively collected.^150^ Other studies suggest the potential of linear measurements of metacarpals applied to radiographs in estimating skeletal sex, yielding accuracy rates of 88–91%.^151^, ^152^ Although promising, caution is advised in the interpretation of the results of these lines of evidence and should not be used as standalone methods. Indeed, further studies are needed to extensively validate their reliability.

Finally, the pelvis, postcranial measurements, and the cranium are the preferred approaches for sex estimation. Most methods that attempt to provide a statistical framework are influenced by inter‐ and intra‐population variations, except for DSP which has been demonstrated to be population independent. Since misclassification can occur, it is, therefore, crucial to use morphological, metric parameters, or discriminant functions standardized to specific reference populations that may better reflect the bones being studied.^138^, ^139^, ^149^, ^153^, ^154^, ^155^, ^156^ Forensic anthropologists have recently addressed the limitations related to the binary nature of sex estimation from the skeleton as a potential barrier to the identification of transgender individuals. The influence of transition on the skeleton is still limitedly understood,^157^ and the identification of signs of gender‐affirming surgery is considerably challenging, especially in cold cases.^158^ The few studies on the topic showed Fordisc is unable to classify transgender women despite extensive gender‐affirming surgery^159^ or found reduced probabilities and typicalities when Fordisc was applied on post‐surgery patients transitioning from male to female.^160^ Although sex classification based on skeletal features typically yields a binary result (male, female, or indeterminate in some cases), forensic anthropologists are advocating for a more inclusive approach. This shift aims to address the complexities of gender identity and protect the rights of marginalized groups, particularly those increasingly targeted by violence.^157^, ^158^, ^161^

#### Molecular

In human identification, commercially available multiplex PCR kits include the amelogenin system, which generates amplicons of 106 and 112 bp for X and Y chromosomes, respectively. However, significant discrepancies have been noted in sex determination using amelogenin due to deletions in the X and Y chromosomes and mutations in primer binding sites. To overcome these issues, all forensic commercial kits have included additional Y chromosome loci, and alternative molecular–genetic assays have been developed.^162^, ^163^ One effective tool for genetic sex determination in a forensic context is based on real‐time PCR amplification of short intergenic sequences (≤50 bp).^164^ With the advent of next‐generation sequencing (NGS) technology, Skoglund et al.^165^ proposed a novel method to evaluate the number of reads in the highly degraded shotgun DNA sequencing data aligning to the X and Y chromosomes. However, this method requires at least 100,000 sequences mapped to the human genome for precise assignment, which is impractical for many poorly preserved samples. To address this, Mittnik et al.^166^ developed an alternative method that evaluates the ratio of sequence alignments to the X chromosome compared to the autosomes, yielding accurate results with just a few thousand reads mapped to the human genome. The use of NGS technology, coupled with these sex estimation approaches, could greatly support forensic cases involving highly degraded bones and/or hair shafts.

#### Integrative approach and interdisciplinary collaboration

The integration of FA and MA significantly improves sex determination in forensic investigations. Forensic methods are well‐established, cost‐ and time‐efficient, and especially reliable for well‐preserved adult remains and when pelvic bones are present. However, there are challenges with juvenile remains, observer variability, and population‐specific parameters. Molecular methods excel in degraded samples and undetermined cases, offering precise and certain results through DNA‐based techniques like PCR and NGS. Combining these methods bridges gaps, and ensures more robust and reliable classification. Interdisciplinary collaboration leverages the strengths of both fields—enhancing forensic outcomes in real‐world scenarios, such as mass disasters, and cold cases. This integrative approach ensures thorough, accurate, and comprehensive sex determination in specific cases where forensic morphometric tools are limited.

### Age at death

#### Forensic

For growing individuals, growth rates in the length of long bones of the diaphysis are efficient parameters for age‐at‐death estimation,^167^, ^168^ as well as metaphyseal and epiphyseal widths, both on bare bone^167^, ^169^ and radiographs.^170^ The rate of development and fusion of primary and secondary ossification centers can be followed in juveniles.^171^, ^172^ Dental development and mineralization are perhaps the most precise indicators in subadults since the growth rate can be followed in the whole dentition or in singular teeth, such as the molars.^173^ A meta‐analysis revealed that the Demirjian method overestimates age by half a year in males and females, still, it is valuable because of the negligible effect of geographical differences.^174^ The London Atlas,^175^ based on radiographs of individuals between 28 fetal weeks and 23 years, is possibly the most updated and straightforward atlas for age estimation of developing subjects.

For adults, the focus is on degenerative changes at selected articular surfaces. The pubic symphysis is the main player in the skeletal age‐at‐death estimation process,^176^ and its use has been extensively validated on specific documented populations.^177^, ^178^, ^179^, ^180^ Most studies on dry bone specimens have yielded variable accuracy rates in age‐at‐death estimation, ranging from approximately 70% to nearly 90% with differences observed between male and female individuals. Some studies have proposed novel traits previously unconsidered that work better for older subjects,^181^ or decision trees that better classify young individuals.^182^ Furthermore, the use of CT images of the pubic symphysis has proved fairly reliable.^183^, ^184^ However, caution is still suggested with imaging tools because of a decreased correct classification rate^185^, ^186^ and in interobserver agreement.^187^

Articular changes at the auricular surface can be evaluated as well. The method has been extensively investigated and tested on other populations with conflicting results, ranging from 80%^188^ and 30%^189^ accuracy rates. Moreover, the method reportedly underestimates and overestimates older and younger age classes, respectively, and thus was even deemed as unreliable when used in isolation for forensic purposes. Revisions to the original method have been proposed,^190^ and subsequent validation studies have yielded mixed results. Some authors have advised caution, showing a weak correlation between the evaluated morphological changes and age‐at‐death^191^ or a general decrease in reliability.^192^ Others, however, have provided more favorable assessments.^193^ Moreover, the combination of the acetabular fossa and the auricular surface^194^ proved to be more reliable, especially in the elderly.^188^ The sternal end of the fourth rib has been criticized over time for its low reproducibility, limited samples, and large age ranges.^195^, ^196^, ^197^ The better performance of Hartnett's^196^ recalibration study on documented samples was confirmed by further tests.^198^ Validation studies were performed on different populations,^199^, ^200^, ^201^ and the research focus is currently moving toward 3D imaging with satisfying results.^202^, ^203^, ^204^

Cranial sutures are often found to be poorly correlated with age in revised methods^205^ or in validation studies.^188^ Recent work supports the need for further investigations^206^ as they cannot be used as a standalone age‐at‐death estimation method in forensic cases because of poor reliability as accurate age indicators.

Multifactorial methods that consider combinations of several articulation sites have been produced. Transition analysis (TA) considers age‐related degenerative signs on cranial sutures, the pubic symphysis, and the auricular surface, providing prior odds from different populations collected in the ADBOU software which calculates likelihood values and 95% confidence intervals for an estimated age.^207^ A few validation tests assessed the accuracy of TA on modern populations and found that cranial sutures showed partially consistent results. In addition to an inconsistent repeatability of the scoring system, large age ranges were recorded impairing the precision of the method^208^ and advancements with respect to traditional methods from individual components. Cranial sutures showed the greater mean absolute error (between 24 and 27), followed by either pubic symphysis^207^ or the auricular surface.^209^ In a new tool which combines 64 traits from seven skeletal districts and neural networks, a mean absolute error of 6 years was produced, significantly improving previous tests.^208^ A validation test recorded higher accuracies in individuals over 50 years old and an improved accuracy of the cranial sutures as age predictors.^210^ However, the poor repeatability and reproducibility of the variables suggest the need for caution in its use and highlight the necessity for improvements.^210^

Dentine transparency has been widely explored, especially in relation to different skeletal population groups, and introduces novel correction factors^211^, ^212^, ^213^ and statistical frameworks.^214^ Cautionary notes have been proposed in respect to buried or burnt teeth.^215^, ^216^ Alternative methods have evaluated the age‐related physiological reduction of the pulp chamber using radiographs.^217^, ^218^ Initially developed by analyzing the surface areas of the pulp chamber and the entire dental element through radiographic studies, these techniques have been refined through further research on multiple teeth and reference populations.^219^, ^220^, ^221^ However, they have faced criticism due to low accuracy rates,^222^, ^223^ or the lack of correlation between age and pulp/tooth ratio.^224^ Recent works have considered the use of 3D models extracted from CT scans to evaluate changes in the pulp chamber volume of canines with promising results, although large prediction intervals were suggested (±30 years with 95% confidence).^225^ Moreover, regression models that take into consideration the degenerative changes in the dental crown and root morphology were produced using 3D models of maxillary teeth, with the maxillary lateral incisor showing the highest correlation with chronological age.^226^

Age‐related changes in the microscopic structure of the cortical bone of the mid‐shaft femur, tibia, and fibula were first investigated by Kerley.^227^ Low intra‐ and inter‐observer agreement was reported in counting osteon and osteon fragments,^228^ thus some studies suggest the use of equations that rely on measurements of osteons and Haversian canals^229^, ^230^, ^231^ so that the method could be applied also to fragmentary remains that present few osteons.^232^

Histomorphometry of ribs has received particular attention with numerous tests on different reference populations,^233^, ^234^, ^235^, ^236^, ^237^, ^238^, ^239^ although the most recent research products on the topic suggest further tests on different reference samples in order to make the method available for forensic investigations.^238^, ^239^

Currently, age‐at‐death estimation for adult individuals primarily depends on observing degenerative changes in articulation sites, particularly the pubic symphysis. Studies on other anatomical regions have shown weak correlations with age or are promising, highlighting the need for further research to refine their application in forensic contexts. Meanwhile, investigations into virtual tools, such as CT imaging, for age‐at‐death estimation are rapidly advancing, though their potential to replace or reliably complement direct observation is still to be fully verified.

#### Molecular

DNA methylation methods are effective tools for age estimation, with a deviation of 3−5 years in the prediction.^240^ Age prediction assays typically depend on bisulfite conversion of template DNA followed by PCR amplification and various sequencing methods. These methods include methylation site‐specific SBE using the SNaPshot kit,^241^ pyrosequencing,^242^, ^243^ EpiTYPERTM technology,^244^ Sanger sequencing,^245^ real‐time methylation‐specific PCR,^246^ and massively parallel sequencing.^247^, ^248^, ^249^, ^250^, ^251^, ^252^ Various statistical approaches have been proposed, including regression modeling methods such as multivariate linear^253^ and nonlinear regression,^254^, ^255^ and neural networks such as generalized regression^247^ and back‐propagation.^253^ Most studies have focused on determining the age of the offender from bodily fluids at the crime scene, while fewer studies have been performed on skeletal remains. The first study on dentin samples was carried out in 2015,^256^ achieving an accuracy of ±4.9 years using the pyrosequencing method. A subsequent study^257^ evaluated DNA methylation in cementum, dentin, and pulp via a MALDI‐TOF mass spectrometry method, with an accuracy between 1.2 and 7.1 years. Another study^258^ measured the methylation patterns of specific genes in teeth and achieved accuracies between 4.8 and 6.9 years.

Researchers have investigated the link between telomere length and chronological age, concluding that telomere length is not a reliable marker for age estimation based on DNA methylation in tooth samples. An alternative model for age estimation, utilizing DNA methylation levels in bones and teeth, has been developed.^259^ This model achieved accuracy of 2.6 years for bones and 11.3 years for teeth using Sanger sequencing, and 7.2 years for bones and 7.1 years for teeth with the SNaPshot assay. Correia Dias et al.^260^ developed two multitissue models incorporating blood, bones, and teeth employing Sanger sequencing technology and the SNaPshot assay. These models demonstrated mean absolute deviations between predicted and chronological age of 6.06 and 6.49 years, respectively. In 2021, the use of DNA methylation for age estimation in pulp tissues was described for the first time, achieving an average absolute deviation of 1.5–2.13 years between predicted and chronological age in adults.^261^ Kondo et al.^262^ tested a new age estimation tool based on methylation levels of DNA extracted from teeth using a real‐time methylation‐specific PCR and found a mean absolute deviation of 8.94 years between predicted and chronological age. Age‐related changes in DNA methylation are highly tissue‐specific,^263^ and many developed models are based on DNA methylation in blood.^253^, ^264^, ^265^, ^266^, ^267^ Therefore, further exploration of CpG sites in bone and tooth samples is needed to refine current models. A recent study on epigenome‐wide DNA methylation at 850 K CpG sites highlighted that 28,549 CpG sites were similarly methylated in bone and blood.^268^

#### Integrative approach and interdisciplinary collaboration

FA relies on growth and aging patterns to estimate age‐at‐death with confidence across an individual's entire lifespan. However, the accuracy of these estimations diminishes in cases involving very elderly individuals, fragmented remains, or populations lacking established reference data. Molecular analysis, particularly through DNA methylation patterns, has shown potential for estimating biological age. However, its application remains limited as most studies have focused on blood rather than bone. This restricts its forensic utility for skeletal remains. Therefore, at present, FA methods alone can reliably estimate age‐at‐death.

While combining forensic and molecular approaches could improve accuracy, current molecular techniques are not yet sufficiently reliable for widespread forensic use in aging skeletal remains and require further refinement. Despite ongoing research in this area, FA continues to be the primary and most dependable method for age‐at‐death estimation. Interdisciplinary collaboration is encouraged, but its effectiveness is currently constrained by the limitations of molecular methods.

### Population affinity

#### Forensic

Craniometric approaches for population affinity estimation are widely explored and employed because of the heritability of the craniofacial metric features that allows a classification in groups.^269^, ^270^ Katherine Spradley and Richard Jantz^271^ improved the data collection methodology suggesting the use of a 3D microscribe that collects landmarks and calculates nonstandard measurements that reportedly perform better. As for analytical support systems, Fordisc^272^ is possibly the most popular analytical program.^273^ Outside of the United States, Fordisc may encounter certain limitations,^274^, ^275^, ^276^ though ongoing updates enhance its applicability in diverse settings. With time, other research groups assessed the efficacy of Fordisc on several populations,^143^ sometimes with unsatisfying results^277^ in different groups compared to the reference. AncesTrees provides another craniometric tool based on a machine learning algorithm,^278^ with an overall high correct classification rate (93%) in the traditional version. When applied to a highly mixed group, rates considerably dropped (48–70%), showing that further integration is needed for its application in cases.^279^

Nonmetric approaches are also extensively employed. The optimized summed score attributes (OSSA) method represents the first statistical framework that empowers classification based on morphoscopic evaluation.^280^ Successful validation tests showed the method to be reliable.^281^ On the downside, the outcomes of the analysis can only be classified into two groups (i.e., Black or White). Caution is, therefore, suggested when applying the OSSA score to samples of different groups. Recently, a decision support system that provides posterior probabilities for a cranium to be classified into four population groups (i.e., European, African, Native American, and Asian) was designed.^282^

Dental metrics provide general geographic patterns (i.e., European, African, Asian) as supporting tools for assessing geographic variation,^283^ as well as frequencies of morphological features of the crown and root.^284^, ^285^ Postcranial morphology^286^ and metrics^272^ have been suggested but still need further validation as it allows only a binary classification (i.e., American White and Black).

Population affinity estimation is arguably the most debated, controversial, and, therefore, unresolved issue in the field.^287^, ^288^, ^289^ Nonetheless, it remains a crucial component of the biological profile aiding in the search for missing persons,^290^ but caution in the interpretation and communication of the results is warranted to avoid possible wrong exclusion when matching profiles.^289^, ^291^ Morphological and metric tools are employed to classify unidentified remains into predefined categories based on reference populations, which inherently affect the estimation outcomes. Morphometric analyses can produce contrasting results,^276^ especially in crania from unreferenced groups, leaving this issue unresolved and requiring future studies. Advances in the field, including the expansion of reference datasets and the inclusion of previously underrepresented populations, are enhancing our understanding of human variation and fostering the development of more accurate methods for forensic applications.

#### Molecular

Based on geographical origins inferred from genetic markers, biogeographical ancestry (BGA) more accurately reflects our focus. BGA can be estimated by analyzing various genetic markers, including short tandem repeats, single nucleotide polymorphisms (SNPs), insertion/deletion polymorphisms (InDels), and microhaplotypes.^292^, ^293^, ^294^, ^295^, ^296^ Autosomal SNPs are particularly effective for BGA estimation due to their stability, widespread distribution across the genome, and significant frequency variation among populations. Since the early 2000s, various ancestry informative single nucleotide polymorphism panels have been developed for forensic ancestry assignment.^297^, ^298^, ^299^, ^300^, ^301^, ^302^, ^303^, ^304^, ^305^, ^306^, ^307^ The SNaPshot minisequencing method (Thermo Fisher Scientific) is commonly used for SNP genotyping in forensics, although it has limited multiplexing capability.^308^ The advent of NGS technologies has enabled the simultaneous genotyping of hundreds of SNPs across multiple samples, leading to the development of new NGS panels by researchers and commercial entities.

The accuracy of ancestry estimation depends on several factors, including the reference populations, the number of markers tested, the informativeness of the markers, the degree of admixture, and the statistical methods employed. Various statistical methods have been used for BGA inference, such as PCA, STRUCTURE,^309^, ^310^ FROG‐kb (http://frog.med.yale.edu/FrogKB/), Snipper (http://mathgene.usc.es/snipper/)—two specific programs designed using the likelihood approach^311^, ^312^—and GenoGeographer.^313^, ^314^ Additionally, multivariate statistical approaches and machine learning techniques have been utilized to identify clusters of genetically structured populations.^315^, ^316^ Discriminant analysis of principal components and partial least squares discriminant analysis (PLS‐DA)/XGBoost have been applied to simulated data and different commercial panels. However, while SNPs can successfully distinguish individuals at the continental level, finer classification remains challenging, presumably because of the limited number of markers in these panels. In 2023, Pilli et al.^317^ developed a new forensic BGA panel with 3234 SNPs and evaluated its ability to infer ancestry using the PLS‐DA method. The results demonstrated excellent discrimination capacity at both intercontinental and intracontinental levels, with variable selection techniques optimizing marker numbers and classification performance. In 2020, Li et al.^318^ introduced a novel approach (SUGIBS) along with an open‐source tool for robust integrative analysis of population structure and genomic ancestry estimation for heterogeneous datasets. Analyzing ancestry informative markers can also provide valuable insights in aged bone samples.^319^ Recently, the BGA of skeletal remains has been inferred using a multidisciplinary approach that incorporates routine forensic markers,^320^ NGS technology,^276^ and forensic skeletal methods.^276^, ^320^

#### Integrative approach and interdisciplinary collaboration

FA uses craniometric and nonmetric techniques to classify individuals based on craniofacial features. However, these methods can be limited by the availability of reference populations and the binary nature of some classifications. MA, on the other hand, uses genetic markers like SNPs, InDels, and microhaplotypes to estimate BGA with higher accuracy at the continental level, though accuracy decreases at the subcontinental level due to the complexity of admixture and reference population limitations.^270^, ^316^ Attempts to integrate the results of FA and molecular methods have so far proven unsatisfactory,^276^, ^320^ making this approach currently difficult to achieve. While molecular methods rely on advanced technology, their integration with forensic techniques has the potential to provide a more comprehensive and accurate framework for analysis. However, this combination remains challenging, particularly given the disparities in methodological reliability and application.

Such an interdisciplinary approach could be especially valuable in contemporary contexts, such as migration crises, where victims are often underrepresented in reference databases. While this perspective may eventually lead to refined methodologies and enhanced forensic investigations, the current state of research suggests that the timing is not yet right for a full merger of these disciplines.

### Stature

#### Forensic

Stature estimation relies on the collection of skeletal metric traits and the application of standardized mathematical equations. When the skeleton is mostly complete, the sum of the elements that contribute to height (the Fully method) can be applied, supported by its revisions,^321^, ^322^ where sex and population affinity do not seem to influence the outcome. With incomplete skeletons, long bones are most frequently used (e.g., femur, fibula, tibia, humerus, radius, and ulna). The dependence on reference populations and secular change have been criticized over time.^323^ This issue remains partially unresolved as some studies suggest that generic formulae efficiently predict stature,^324^, ^325^ whereas recent tests support the need for sex and population‐specific equations.^326^, ^327^ Moreover, Ousley suggests the use of prediction intervals rather than regression equations.^141^ Fordisc^272^ automatically calculates stature estimation according to several reference groups, with limitations related to the reference groups. Stature estimation from other skeletal districts,^328^ fragmentary remains,^329^, ^330^ and subadult individuals^331^, ^332^, ^333^ has been investigated, although caution is generally suggested.

#### Molecular

Human height is a complex trait influenced by multiple genetic variants. Genome‐wide association studies (GWAS) have pinpointed several genetic variants linked to adult human height. Two such studies were published in 2008,^334^, ^335^ and in 2009, Aulchenko et al.^336^ attempted to predict height by analyzing 54 height‐associated SNPs in a thousand Dutch individuals. In 2021, Lanktree et al.^337^ conducted a meta‐analysis of 47 GWAS involving 114,223 adults from six ethnicities and identified 64 loci significantly associated with height. The Genetic Investigation of ANthropocentric Traits (GIANT) consortium, in 2010, identified 180 loci significantly associated with height through a GWAS study of over 180,000 individuals and nearly three million SNPs.^338^ However, these 180 polymorphisms explained only about 10% of height variation, while twin studies estimate the heritability of height to be around 80%.^339^ Additional studies have positively investigated the association of some of these SNPs with the height of children and adolescents in different populations.^340^, ^341^, ^342^, ^343^ Since height is influenced by various mechanisms, both environmental and genetic, which change from infancy to early adulthood, Paternoster et al.^344^ examined the genetic influence on childhood growth. Their results showed that the 180 known adult height SNPs were consistently associated with growth from 1 to 10 years. Liu et al.^345^ also studied the 180 adult height SNPs, focusing on tall individuals, and found that 166 out of 180 normal height‐associated variants (i.e., 96%) were also involved in determining the tall stature of Europeans with prediction accuracy considerably improved compared to a previous attempt. In a study involving 253,288 individuals, the GIANT consortium identified 697 DNA variants linked to adult height in Europeans,^346^ which together explained about one‐fifth of the heritability for this trait. When these markers were tested in a non‐European population, the prediction accuracy was lower compared to Europeans.^347^ Despite the small individual effect of each variant,^348^ their combined influence can be highly predictive, as suggested by Yengo et al.^349^. A meta‐analysis of approximately 700,000 individuals of European ancestry showed a significant increase in the number of loci associated with height compared to previous GWAS analyses. However, Sohail et al.^350^ caution against overinterpretation of these findings. To explore the evolution of complex traits, both single‐locus studies^351^ and genome‐wide analyses^352^, ^353^, ^354^ have been conducted on ancient bone samples. Cox et al.^355^ compared genetic predictions with actual stature measurements from skeletons and found discrepancies in some cases, highlighting the need for caution when interpreting genetic predictions without direct phenotypic measurements.

#### Integrative approach and interdisciplinary collaboration

FA relies on measurements and regression equations to estimate stature, which is effective for well‐preserved remains. However, the condition of the remains and reference population representativeness can limit accuracy. Molecular methods use genetic markers identified through GWAS to predict stature, even from degraded DNA. However, the predictive power of genetic markers is still limited and only accounts for part of the variation in height. While combining these methods could expand the biological sources for stature estimation, molecular techniques remain untested on bones and are not yet reliable for forensic use. Collaborative efforts between forensic and molecular anthropologists could improve the accuracy and reliability of stature estimation in the future by integrating both morphological and genetic data.

### Soft tissue features (hair, eyes, skin)

#### Forensic

Nonskeletal biological evidence is not uncommon in forensic cases,^356^ although such an occurrence is not guaranteed in every investigation. Forensic anthropologists may collaborate with, and seek expert opinions from, other forensic specialists to analyze skin appendages such as hair and nails. In particular, the identifying potential of the ridge pattern of nails has been investigated in a few studies.^357^, ^358^, ^359^ Similarly, individualizing tattoos, scars, and moles can be detected in decomposed skin sometimes related to skeletal remains that should be sent to the forensic pathologist for proper evaluation.^360^, ^361^ Such information, however, cannot be derived solely from skeletal remains.

#### Molecular

On the other hand, MA can provide interesting information on the aspects of a person from external soft tissue (e.g., skin). The prediction of an individual's phenotypic traits is another key component that can be used for the biological profile of human remains.

##### Eye, skin, and hair color

Forensic DNA phenotyping, which predicts physical traits such as eye, skin, and hair color from DNA, has advanced significantly thanks to GWAS and a deeper understanding of human genome variation. The IrisPlex system was the first method to predict eye color using six DNA variants and a multinomial regression model.^362^, ^363^ Its successor, HIrisPlex, expanded to predict both eye and hair color by analyzing 24 DNA variants with two multinomial regression models.^364^ HIrisPlex has been successfully used to analyze DNA from bones and teeth of various ages and conditions,^365^ including World War II skeletal remains,^366^ bones attributed to King Richard III of England,^367^ and a femoral bone attributed to Jörg Jenatsch.^368^ An 8‐SNP multiplex assay was also developed and validated^369^ for challenging samples, including bones. More recently, the HIrisPlex‐S method was developed,^370^, ^371^ analyzing 41 DNA variants and using three different algorithms to calculate probabilities of eye, hair, and skin color categories. This method includes a user‐friendly web interface (https://hirisplex.erasmusmc.nl/) that provides prediction probabilities for three eye color, four hair color, and five skin color categories based on 41 SNPs. Initially developed for the SNaPshot methodology, these methods have transitioned to NGS technology allowing for the analysis of all 41 HIrisPlex‐S markers in a single reaction^372^ and the development of new assays with more markers for appearance and ancestry inference.^373^ The HIrisPlex‐S MPS assay was also validated on bone samples with varying levels of DNA degradation.^374^ In recent years, DNA‐based prediction of eye, hair, and skin color has become a standard approach in forensics,^375^ with several statistical models developed for this purpose.^376^ Despite the rise of machine learning methods for classification and clustering,^377^ multinomial logistic regression remains the preferred method over the three machine learning methods investigated (support vector machine, random forest, and artificial neural networks) for predicting pigmentation traits from DNA. Katsara et al.^378^ examined the impact of trait prevalence–informed priors on Bayesian models predicting eye color, hair color, skin color, hair texture, and freckles and found that these priors can enhance model performance but require careful definition of such priors.

##### Hair morphology

As well as other phenotypic traits, the shape of the hair follicle and consequently the hair morphology (i.e., straight, wavy, and curly) is also genetically determined. Although the genetic factors contributing to hair shape are not yet fully understood, Pośpiech et al.^379^ conducted a genetic investigation on head hair morphology. They developed the first prediction model based on three SNPs in a sample of 528 Europeans, achieving a prediction accuracy with an area under c urve (AUC) of 0.622. Later, a new prediction model using 14 SNPs was created, showing prediction accuracies of 0.66 and 0.64 in 6068 Europeans and 977 independent Europeans, respectively.^380^ Additionally, a binomial model utilizing 32 SNPs was developed to predict hair shape,^381^ achieving accuracy rates of 0.664 for Europeans and 0.789 for non‐Europeans. In 2020, Ho et al.^382^ conducted the first GWAS focusing on a micro‐level quantitative measure of hair curvature, aiming to identify additional genomic loci that influence this trait. This research expanded on Liu et al.’s earlier GWAS,^383^ which employed digital methods to quantify continuous variations in human eye color. Early studies indicated an overlap between the genetic markers involved in various hair phenotypes.^379^, ^384^, ^385^, ^386^, ^387^, ^388^ To further explore the genetics of these phenotypes, Pośpiech et al.^389^ analyzed 240 SNPs in a population of 999 Polish individuals and found a significant number of shared loci (approximately 15%) for hair‐related features, as well as pleiotropy and epistatic effects in determining these characteristics. The omnigenic model of complex traits proposed by Boyle et al.^390^ could explain these results. Understanding the genetics of hair morphology, which varies within and among populations, could enhance phenotypic studies and lead to new forensic DNA phenotyping models. These models could include genetic markers for hair shape, baldness, and beard thickness, providing a more accurate characterization of individuals.^391^

##### Baldness

Baldness, or androgenetic alopecia, is a prevalent condition marked by partial or complete hair loss. This condition is strongly associated with male sex hormones and genetic predisposition, with an estimated heritability of 80%.^392^ Among the various loci potentially involved, five genes, some of them directly linked to the production of androgen receptor and ectodysplasin A2 receptor, were identified as the best predictors. These genes were used to develop an initial predictive model for male pattern baldness (MPB), achieving an accuracy of 76.2%.^393^ Adding 15 SNPs to this model increased the prediction accuracy to 86.4%, illustrating that even markers with lower predictive power can enhance the prediction accuracy when combined with stronger ones.^393^ Another predictive model, incorporating 14 SNPs, was developed^394^ and demonstrated a 74% accuracy in predicting early‐onset MPB in a sample of 2725 German and Dutch males. Further research^395^ identified over 250 genetic loci associated with severe hair loss using data from 52,000 English male participants aged 40–69 years. This led to the development of a prediction algorithm capable of distinguishing individuals without baldness from those with severe hair loss. Additional studies^396^, ^397^, ^398^ have identified 63, 71, and 624 genetic loci associated with severe hair loss. Chen et al.^399^ have developed the most reliable genetic prediction models for MPB so far, leveraging numerous genetic predictors and extensive, independent datasets. Despite this progress, the study indicated that achieving highly accurate genetic prediction of MPB is still a challenge.

#### Integrative approach and interdisciplinary collaboration

FA cannot predict phenotypic traits such as eye, hair, and skin color, unless found in association with the remains. Among the variables, the condition of the remains strongly affects the possibility to gain information about these features. MA complements this by analyzing degraded DNA to predict soft tissue features with high accuracy using tools like the HIrisPlex panel. By combining morphological data from FA with genetic predictions from molecular methods, experts can develop a more detailed and accurate biological profile. This integrative approach enhances the matching process with missing people, provides valuable insights into both victims and offenders, and paves the way for innovative forensic investigations. While FA excels in skeletal analysis, MA fills the gap in predicting external characteristics, making their collaboration essential for more effective and reliable forensic work.

### Craniofacial features

#### Forensic

After reconstructing the biological profile and examining the skeleton for dental work or anomalies, bone calluses or other pathologies may contribute to a facial reconstruction, or an approximation can be attempted. Although this is not an identification method, and it is not a routine aspect of forensic anthropological analyses, it may represent a last resort tool contributing to the process.^400^ Originally pioneered by Gerasimov,^401^ facial reconstruction can be performed either manually on a dry skull, skull cast,^402^ or via computerized techniques. Soft tissue thickness is still being investigated for the development of population‐specific studies and reproducibility tests.^403^, ^404^, ^405^, ^406^ The introduction of the free and open repository CRANIOFACIALidentification.com provides a novel set of data on facial soft tissue depth data.^407^ Advances in computer technology provide a powerful tool for facial approximation, shifting the discipline toward novel electronic devices that quicken manual techniques.^400^, ^408^, ^409^, ^410^, ^411^, ^412^

Accuracy results are very diverse, but one should keep in mind that the outcome of the facial approximation is solely a recognition aid (i.e., trigger) that may suggest a resemblance between the missing and the unidentified body,^413^ rather than an accurate tool to reproduce facial features. The reader is redirected to Stephan et al.^414^ for an up‐to‐date and comprehensive overview on facial imaging that exhaustively summarizes the history and recent developments of the discipline.

#### Molecular

Genetic variation plays a significant role in determining facial morphology,^415^, ^416^ as evidenced by the high resemblance seen in monozygotic twins. The human face is a complex morphological structure influenced by a combination of genetic, environmental, and cellular factors.^417^, ^418^, ^419^ Numerous GWAS studies^386^, ^387^, ^420^, ^421^, ^422^, ^423^, ^424^, ^425^, ^426^, ^427^, ^428^, ^429^, ^430^, ^431^, ^432^, ^433^, ^434^, ^435^, ^436^ have identified over 100 loci associated with normal‐range facial morphology. For instance, White et al.^437^ identified 203 genome‐wide–significant signals linked to facial variation, while Naqvi et al.^438^ found 76 genomic loci related to face shape in 19,644 Europeans. A recent genome‐wide association analysis^439^ evaluated 20 quantitative facial measurements in 2447 European individuals and identifies several suggestive variance quantitative trait loci. Since most loci have been identified in Europeans, Liu et al.^440^ explored the genetic basis of facial morphology in an East African cohort and revealed significant signals at 20 loci, 10 of which were shared with Europeans. Bonfante et al.^441^ conducted a GWAS on over 6000 Latin American individuals and found significant associations with 32 traits. Additionally, Null et al.^442^ performed a GWAS using copy number variant (CNV) markers in 3388 Bantu African children, highlighting the role of CNVs in determining facial morphology. Sex and ancestry significantly influenced normal craniofacial variation. Genomic ancestry alone accounts for 9.6% of the total facial variation, while sex independently contributes 12.9%.^443^ Similar results were observed by Lippert et al.^444^, who performed whole genome sequencing on 1061 individuals from diverse ancestral backgrounds. Despite the interest in reconstructing facial features via DNA analysis for investigative purposes or cold case reviews, it is currently not feasible to use genomic data to create an accurate facial sketch.

Parabon Nanolabs (https://parabon‐nanolabs.com/) has applied machine learning to predict phenotypic traits and identify associations across a vast collection of DNA samples and facial images.^445^ However, the company has not yet published any methodology or validation tests.

#### Integrative approach and interdisciplinary collaboration

FA should use facial reconstructions (either manually or virtually produced) only as a trigger, rather than an exact reproduction of facial features for identification. Molecular analysis focuses on genetic variation, with GWAS identifying loci linked to facial morphology. Despite progress, using genomic data to accurately reconstruct facial features remains unfeasible due to the complexity of facial traits influenced by multiple factors. FA benefits from technological advancements, although the outcome is limited by the condition of the remains and the imprecision of facial reconstruction. Molecular methods, while informative about genetic influences on facial features, cannot yet provide detailed facial reconstructions from DNA alone. The complementary use of these methods is still challenging, as molecular techniques are not yet advanced enough to replace traditional forensic methods. However, integrating both fields through interdisciplinary collaboration can improve craniofacial reconstructions over time, but only to match possible candidates and not to positively identify.

### Pathological markers

#### Forensic

Although pathological bone markers are not usually included in the traditional four pillars of the biological profile, they represent highly informative factors for the health status of the individual. Therefore, pathological conditions observed on the remains can significantly complement the biological profile^95^, ^446^ and provide the anthropologist with individualizing features to employ in finding matches among missing persons and in the following steps of personal identification.^95^, ^96^ AM trauma, bone calluses from fractures, and prosthetic devices offer crucial insights into an individual's health status, access to healthcare,^447^ and even facial appearance (e.g., a broken nose), posture, and gait (e.g., fractures of the limbs).^96^ Moreover, these features can also serve as valuable factors in personal identification,^96^, ^447^ although their uniqueness in the context under study should be thoroughly assessed.^29^, ^35^, ^36^

The lack of skeletal evidence for certain diseases does not necessarily mean the individual was healthy, as not all pathologies leave detectable marks on bones.^448^ Stress markers,^449^, ^450^, ^451^, ^452^, ^453^ as well as signs of congenital^454^ and multifactorial diseases,^455^, ^456^, ^457^ are recent topics of investigation, especially for their individualizing potential. Information on the health status of the deceased may also derive from nonskeletal biological evidence, such as vascular calcifications.^458^, ^459^

A recent shift in the definition of the biological profile in FA involves viewing specific lines of evidence as a product of marginalization and discrimination. A whole section of *Forensic Science International: Synergy* in 2023 was dedicated to this emerging topic.^460^, ^461^, ^462^, ^463^, ^464^, ^465^, ^466^, ^467^ The Structural Vulnerability Profile (SVP) examines how social, economic, and environmental inequalities heighten an individual's risk of experiencing violence, injury, or harm. SVP bridges FA and social science, recognizing that an individual's exposure to risk—and potentially the circumstances of their death—is influenced by structural inequalities such as poverty, marginalization,^464^ racial discrimination,^465^ gender‐based violence,^157^, ^468^ and limited access to resources.^467^ By integrating this perspective, forensic anthropologists can shed light on the lived experiences of individuals and communities, often revealing the deep impact of structural inequalities.

#### Molecular

Recent advances in high‐throughput technologies have opened the possibility not only to identify from bone samples the pathogenic microbiome useful for understanding causes of death, such as screening of *Mycobacterium tuberculosis* in 28 skeletal remains from central Poland,^469^ but also to investigate individual predisposition/susceptibility to develop the disease in order to provide more information to characterize the biological profile (e.g., see Refs. 470, 471).

#### Integrative approach and interdisciplinary collaboration

FA provides valuable insights into the health of an individual through the detection of a wide spectrum of pathologies and traumatic events. However, not all diseases leave detectable traces on bones, and some health conditions may remain invisible. Molecular analysis and leveraging advancements in high‐throughput technologies allow for the identification of pathogenic microbiomes and genetic predispositions to disease, offering a deeper understanding of an individual's health and cause of death. The strengths of FA lie in its ability to detect visible pathological markers, but it is limited by the preservation state of the remains and the absence of skeletal evidence for certain diseases. Molecular methods successfully identify pathogens and genetic predispositions, although they cannot predict trauma, bone healing, or the development of a disease due to the influence of environmental and lifestyle factors. Combining these approaches enriches the biological profile by integrating morphological data with genetic information, providing a more holistic view, but it requires further research to optimize its use in forensic contexts.

## CONCLUSIONS

The identification of skeletal remains is a complex and vital task in forensic science, serving civil, criminal, and administrative purposes. The conditions under which bodies are discovered often influence this process, frequently leaving the skeleton as the primary, and sometimes sole, source of information. FA and MA are rarely integrated during investigations, although the collaboration between the two fields could lead to significant advancements and improvements for both the investigation and the discipline.

**FIGURE 1:**
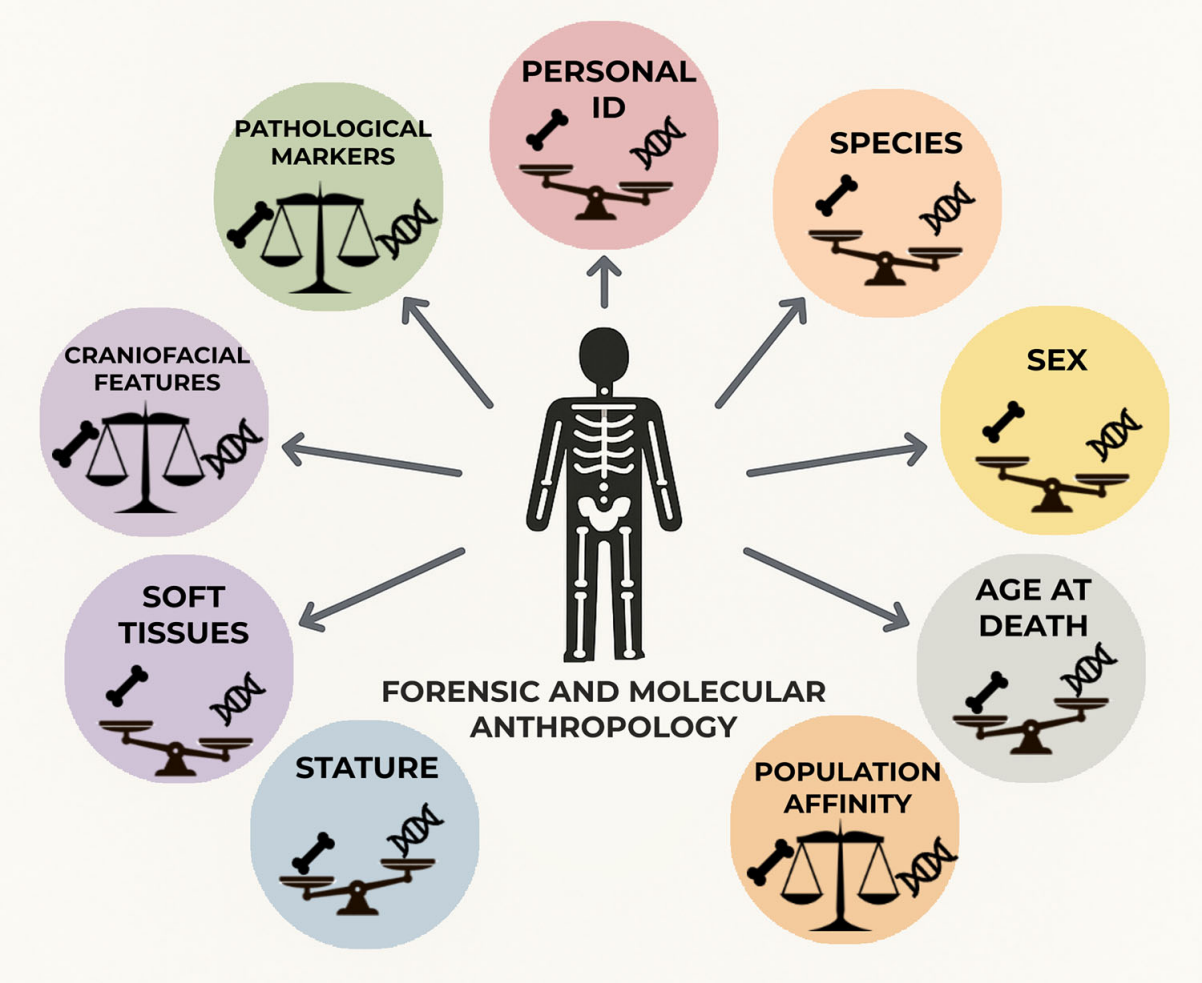
Graphical representation of the respective contributions of forensic anthropology (represented by a bone) and molecular anthropology (represented by a DNA double helix) to personal identification and biological profile. The scale is shown tipping in favor of either molecular or forensic anthropology. In some situations, the scale tips in favor of molecular anthropology (species ID, sex estimation, soft tissues feature). Some situations favor forensic anthropology (age at death and stature), while in other cases, it is balanced (population affinity, craniofacial features, pathological markers).

Positive identification remains the primary objective in forensic investigations involving unidentified skeletal remains. Among the various methods available, DNA analysis stands as the most reliable tool, offering unmatched precision in linking remains to specific individuals (Figure 1). Regardless, its effectiveness can be limited in certain contexts, particularly when reference samples are unavailable. However, if genetic identification cannot be achieved, many times morphological identification can be sufficient and replace DNA identification, so long as the skeletal or dental traits visible in the AM and PM data are present in number and quality, which make them reliable indicators of uniqueness. In recent years, FA has even begun to investigate the potential of statistical tools to enhance morphological identification, particularly in achieving error quantification in the same manner as DNA. This approach represents a promising step forward for the discipline by potentially bridging the gap between traditional anthropological methods and DNA‐based identification. Such advancements could enhance the juridical usability of morphological analysis and bringing it closer to the reliability of DNA evidence and further strengthening the identification process.

The integration of evidence from both FA and MA into a unified identification system remains an underexplored yet intriguing possibility. Current practices often rely on combining evidence from both disciplines, resulting in identifications that are statistically validated primarily by DNA. However, this approach overlooks the potential of combining multiple identity indicators from different disciplines in a systematic way. Imagine a scenario where an algorithm is designed to assess the reliability of an identification by synthesizing evidence from both morphological traits and DNA markers. Such a system could revolutionize the field by offering a robust, statistically supported framework for positive identification. By weighing and integrating data from multiple sources, this approach could provide the most reliable and conclusive evidence possible, and set a new standard for forensic investigations.

DNA is a sound technique to determine the human or nonhuman origin of a fragment, although less cost‐ and time‐efficient than microscopy (Figure 1). The chosen approach should, therefore, consider the extent of fragmentation, cost, and time. Therefore, samples should be preserved for both forensic and molecular analyses in case one discipline fails to provide accurate results. Skeletal sex is certain through DNA, although it can be directly and reliably determined using FA when the pelvis is present. In contrast, other portions found in isolation (e.g., the cranium or long bones) may not be as reliable, hence the need for DNA analysis. Age‐at‐death and stature are undeniably prerogatives of FA (Figure 1) despite recent advancements of MA which are at present not reliable enough. Population affinity remains the most debated aspect of the biological profile for both FA and MA. So far, the literature has not found a way to integrate the results (Figure 1).

Unless well‐preserved and found in association with the skeletal remains, FA cannot reliably predict soft tissue features, whereas MA is able to accurately complete this task thanks to recent advancements in the field (Figure 1). Facial reconstructions and features derived from DNA are only to be used as a trigger for people looking for a missing person but are not identification methods. As for pathological markers, the benefits of FA and MA are nearly equal. FA can recognize a wide array of pathologies based on bone anomalies and MA is extremely valuable for detecting pathogens or predisposition and may assist and confirm the interpretation of some pathological signs (Figure 1). Although not directly related to FA and MA, it is worth mentioning that research is calling attention to the potential of skeletal remains as a source of toxicological information, which can prove extremely useful in biological profiling.^95^, ^472^, ^473^

In conclusion, each forensic case presents its own set of unique challenges and is shaped by a variety of unpredictable factors that dictate whether a forensic or molecular approach is more appropriate (also depending on costs, time, facilities, and equipment). Flexibility and an open mind are essential as the combination of both disciplines can be highly beneficial. By integrating forensic and molecular methods, each can compensate for the limitations of the other, ultimately enhancing the accuracy of identification efforts and providing either a more comprehensive and detailed biological profile or the identification of the individual.

## AUTHOR CONTRIBUTIONS

E.P. and C.C. conceived the idea of a comparative approach. E.P. dealt with aspects of molecular anthropology. A.P. and C.C. focused on the forensic anthropology aspects. All authors contributed to the writing and editing process.

## COMPETING INTERESTS

The authors declare no conflicts of interest.
